# PET imaging for receptor occupancy: meditations on calculation and simplification

**DOI:** 10.1016/S1674-8301(12)60014-1

**Published:** 2012-03

**Authors:** Yumin Zhang, Gerard B. Fox

**Affiliations:** Translational Sciences, Global Pharmaceutical R & D, Abbott Laboratories, Abbott Park, IL 60064, USA.

**Keywords:** positron emission tomography, receptor imaging, receptor occupancy, drug development, central nervous system

## Abstract

This invited mini-review briefly summarizes procedures and challenges of measuring receptor occupancy with positron emission tomography. Instead of describing the detailed analytic procedures of *in vivo* ligand-receptor imaging, the authors provide a pragmatic approach, along with personal perspectives, for conducting positron emission tomography imaging for receptor occupancy, and systematically elucidate the mathematics of receptor occupancy calculations in practical ways that can be understood with elementary algebra. The authors also share insights regarding positron emission tomography imaging for receptor occupancy to facilitate applications for the development of drugs targeting receptors in the central nervous system.

## INTRODUCTION

The interaction of a receptor and its ligand is highly selective and this specific interaction is the primary mechanism of drug activity within the central nervous system (CNS). The relationship between drug dose level and the percentage of receptor occupancy [*O*(%)] achieved is a measure critical to the development of a CNS drug candidate, regardless of whether it is an agonist or a competitive antagonist. Receptor imaging with a radiolabeled ligand using position emission tomography (PET) is a powerful technique that can be used to non-invasively measure receptor occupancy in humans. Without the need to radiolabel the drug itself, receptor occupancy studies using PET imaging can answer critical questions, such as whether the drug has entered the brain, engaged its target receptor, and whether the dose level achieves sufficient receptor occupancy level for desired effects at an adequate therapeutic index.

However, receptor occupancy calculation methods published in the scientific and medical literature are often too general to provide operational guidance, or overstated with confusing mathematic manipulations, which tend to scare away general researchers. We herein intend to introduce a pragmatic overview for receptor occupancy measurements and simplified calculations.

## PET TECHNOLOGY

**Fig. 1:**
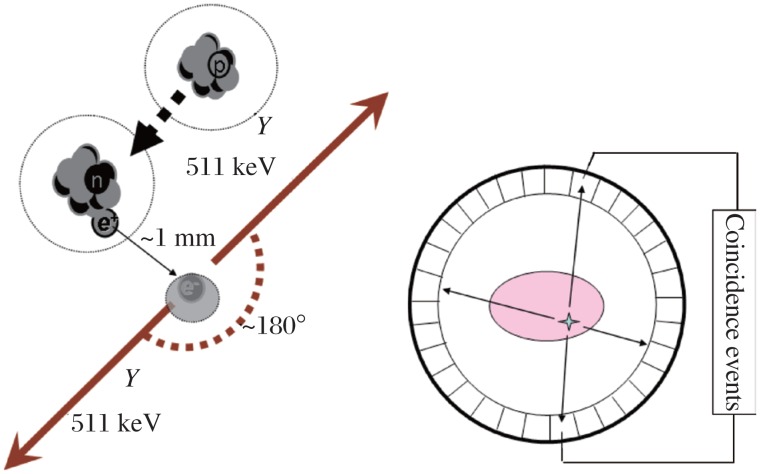
The principles of positron annihilation and positron emission tomography.

Positron emission tomography stands for position emission tomography (PET). Positrons are the antiparticles of electrons that are emitted from the nucleus of some cyclotron-generated radionuclides. When a positron-emitting radionuclide decays, a positron (positively-charged electron) is emitted from the nucleus and almost immediately collides with a nearby orbital electron. After the collision and subsequent annihilation, two 511 keV gamma ray photons are produced emitting 180° apart from each other. This emission can be detected by a PET scanner with an array of coincidence detectors. When a positron-emitting radionuclide labeled compound is introduced into the body as a tracer (or a radioligand to a receptor), the tracer reports back from the body as the emission signal to indicate the location of the labeled compound. PET imaging uses these signals to reconstruct the tracer's *in vivo* distribution as three dimensional tomography (tomo- in Greek means slice) (***Fig. 1***).

In theory, PET can track *in vivo* movement and distribution of any molecules as long as the molecule can be radiolabeled by a positron-emitting radionuclide such as ^11^C (T_1/2_ = 20 min) and ^18^F (T_1/2_ = 110 min). After introduction into the body, most often intravenously, the tracer is subjected to distribution, accumulation to target tissue, metabolism and clearance. Selective target accumulation reflects the specific molecular interaction of the tracer and its target. A dynamic PET imaging study can, if needed, track the distribution of a positron-labeled compound at any body region over time and provide tissue pharmacokinetics as time-activity curves. For neuroreceptor imaging, PET imaging focuses on the brain, and in this case, the tracer is a radiolabeled ligand.

### Design of PET receptor occupancy studies

**Fig. 2:**
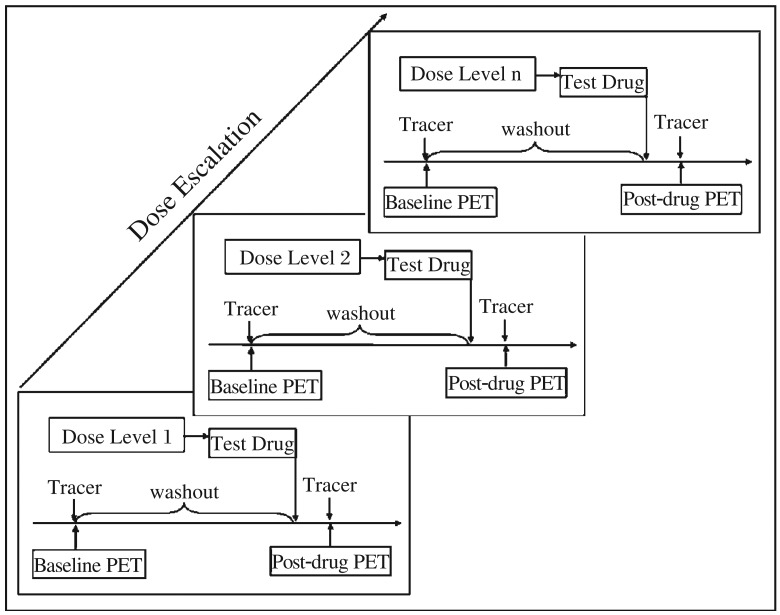
Design of receptor occupancy studies using positron emission tomography imaging to evaluate a test drug.

The calculation of receptor occupancy [*O*(%)] is based on the measured reduction of the specific tracer uptake from the baseline due to the occupancy of receptor binding sites by a given dose of the drug under investigation. To achieve this, two PET scans are performed on each subject in the sequence of: baseline PET (before drug administration) followed by postdrug PET (after drug administration) (***Fig. 2***).

In a baseline PET imaging, only the radioligand is given to measure selective uptake and accumulation into receptor-rich regions of the brain. Followed by administration of the test drug, the tracer is given again and a second PET scan is then performed as the post-drug PET. Reduction of radioligand uptake in the post-drug PET in comparison to the baseline PET is used to determine receptor occupancy [*O* (%)]. Each pair of baseline PET-post-drug PET scans will provide one [*O* (%)] value.

**Fig. 3:**
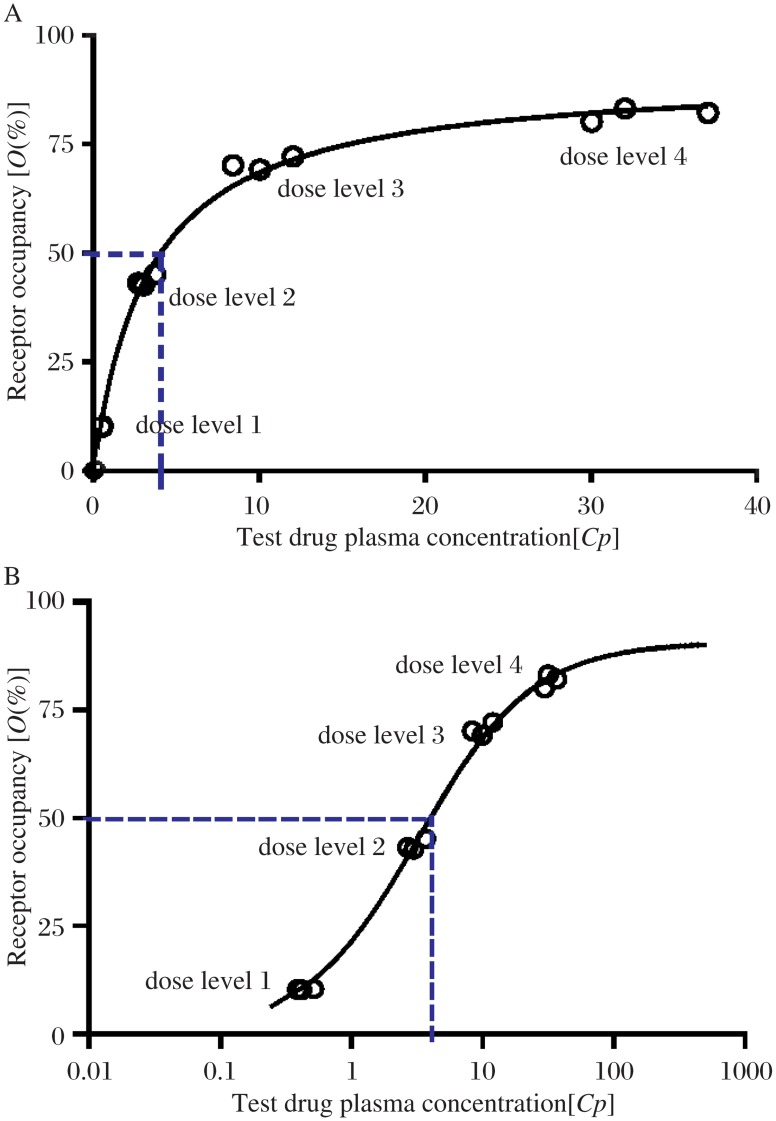
The receptor occupancy curve reveals the relationship of the receptor occupancy [*O* %] levels and the required test drug plasma concentrations [*Cp*] expressed in linear (A) and log scale (B).

To obtain a comprehensive picture of the receptor occupancy of a test drug, it is advisable to plan at least 4 cohorts of subjects. Each cohort of at least three subjects represents one dose level, which is escalated within the maximum tolerated dose obtained during phase 1 dose-escalation clinical studies (***Fig. 2***). When individual [*O* (%)] values are plotted against corresponding drug dose levels or plasma concentrations of the test drug, a receptor occupancy curve can be generated (***Fig. 3***).

### Calculations of receptor occupancy [*O* (%)]

The formula used for the calculation of receptor occupancy [*O* (%)] is quite simple as shown[1]: 
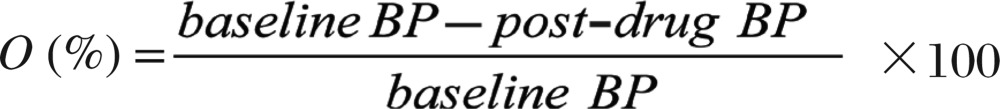
(1) Where BP is the binding potential of the receptor and its ligand.

From the equation (1), the task of receptor occupancy imaging is to obtain the binding potential values of PET imaging, before and after administration of the test drug. Because the measurement of binding potential is not straightforward, it will be helpful to provide a more detailed explanation for better understanding of binding potential.

### Binding potential

Binding potential is introduced for PET imaging, based on *in vitro* radioligand binding experiments, as a pivotal measure to calculate the receptor occupancy *in vivo*[2],[3]. Binding potential is a combined measure describing the ultimate binding force of a receptor to its ligand, denoted as the product of maximum density of a neuroreceptor (B_max_) and the affinity of a specific ligand to its receptor (i.e. binding potential = B_max_ · affinity). As some receptors are occupied by endogenous ligands, the B_max_ here denotes the available receptor density. Because affinity of ligand binding is the inverse of K_D_ (radioligand equilibrium dissociation constant), the binding potential is expressed as binding potential = B_max_/K_D_[2].

The following section describes how binding potential in PET receptor imaging is derived from *in vitro* radioligand binding.

**Fig. 4:**
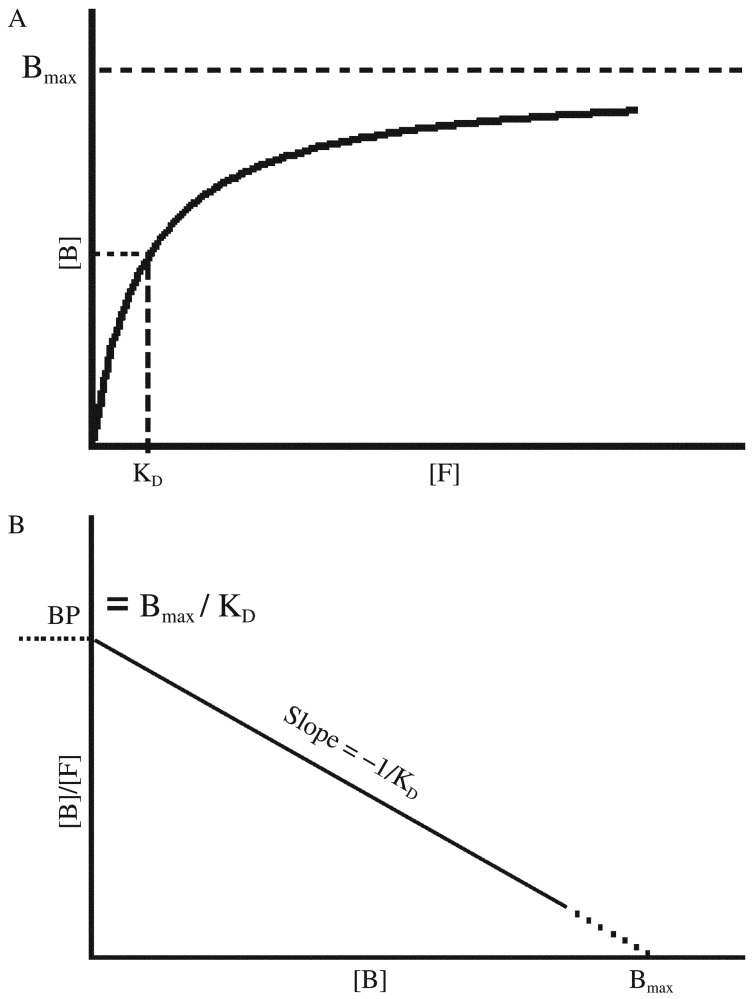
Receptor-binding assay expressed as non-linear curve fitting (A) and transformed into linear regression as Scatchard plot (B) for binding parameter estimation.

By the law of mass action, the concentrations of receptor-bound ligand ([B]) have the following relationship when at equilibrium with the concentrations of free ligand ([F]): 
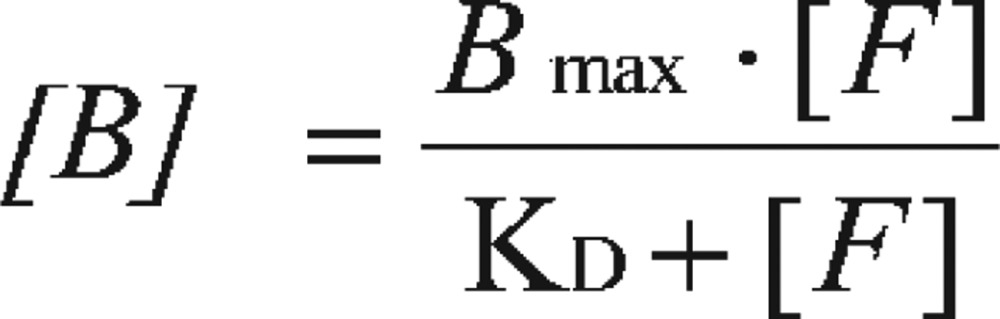


A saturation curve can be generated by plotting concentrations of receptor-bound radioligand ([B]) against the increasing concentrations of free radioligand ([F]) (***Fig. 4A***). By nonlinear regression fitting, the B_max_ and K_D_ can be obtained, with K_D_ representing the concentration of free ligand when ½ B_max_ is reached. However, in the days before nonlinear regression programs were widely available, the data needed to be transformed into a linear form for analysis by linear regression. The most popular method to linearize binding data is to create a Scatchard plot (***Fig. 4B***) that is expressed by the linear equation: 
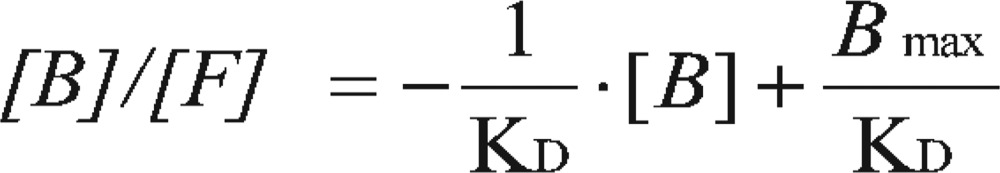


In this plot, the X-axis represents specific binding, bound, or [B]. The Y-axis represents specific binding divided by free radioligand concentration, i.e. Bound/Free, or [B]/[F].

From this equation, at very low radioligand concentrations, there will be very limited receptor binding, i.e. when *X* = [*B*] = 0 
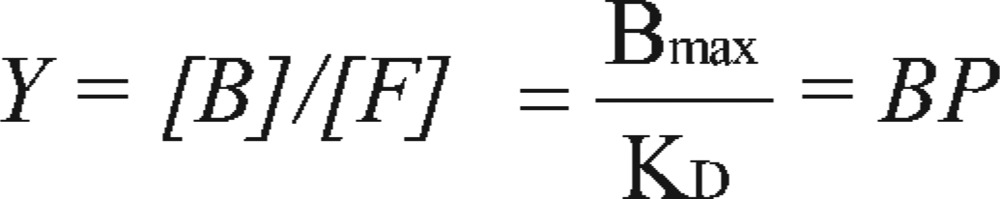


Therefore, the binding potential is the Bound/Free ratio ([B]/[F]) when the ligand is at a very low concentration.

*In vivo* PET studies utilize tracer technology with very low concentration of radioligand, occupying only a small fraction of the available receptors (i.e. Bound → 0). Therefore, without the luxury of conducting a study with increasing concentrations of radioligand to generate a Scatchard plot, for obtaining binding potential by B_max_/K_D_, PET imaging can directly measure the binding potential value as Bound/Free, the ratio of specifically bound ligand concentration (C_B_) to free ligand concentration (C_F_):

Binding potential = B_max_ / K_D_ = C_B_ / C_F_

The goal or the challenge of PET receptor imaging is to measure the binding potential or the ratio of C_B_ over C_F_ in the target region, i.e. the receptor-rich region.

However, it is almost impossible to distinguish C_B_ and C_F_ within the same region *in vivo*. Thus, an important assumption needs to be introduced, that, at equilibrium, the free ligand concentration in tissue (C_F_) equals the ligand concentration in plasma (C_P_), when a radioligand is transported into brain tissue freely by passive diffusion[2],[4].

Binding potential = B_max_ / K_D_ = C_B_ / C_F_ = C_B_ / C_P_

The C_P_ can be measured directly from arterial blood samples.

The following section of this review will focus on how PET imaging answers the challenge of obtaining binding potential or C_B_ noninvasively with a single imaging.

### PET procedure for receptor occupancy [*O*(%)]

The PET procedure for receptor occupancy [O (%)] estimation is much more complex than PET imaging for diagnosis of disease. It will be helpful to summarize the procedures of these kinds of experiments for new researchers to better design and conduct their own PET imaging studies.

#### Preparation of positron-labeled radioligand

A radioligand or tracer is prepared by labeling a compound with a position-emitter. The tracer usually has been well validated. Otherwise, an extensive validation is required for a novel tracer. The latter always has an in-depth description of the chemistry and radiochemistry of preparation and quality control to ensure the injectability for human use. The precursor of the tracer is rarely selected from screening. Instead, it usually comes from one of the drug candidates possessing the selective targeting properties that can be used as radioligand for PET imaging with a reasonable target over non-target contrast. Detailed attributes of a PET ligand can be found elsewhere[4]. The subjects used for this kind of study are healthy human volunteers or patients, and sometimes non-human primates are used for early stage drug development.

#### Tracer dosing and PET image acquisition

The tracer is typically administered intravenously. Tracers are most commonly delivered as bolus, but sometimes are dosed as bolus followed by continuous infusion. The manner of administration depends on the kinetics of the tracer and its interaction with the receptor. Subjects are placed on the PET imaging bed in the supine position with his/her head extending into the detector ring of PET scanner. The images are taken with pre-determined acquisition parameters. Raw PET imaging data are acquired in list-mode format, recording the annihilation events during the radioactivity decay from the positron-nuclide attached to the tracer. When a dynamic PET scan is performed, PET data are acquired from the time of injection to the time when all the important changes in tracer kinetics have been observed.

Meanwhile, serial arterial blood samples are often collected to measure the parental tracer plasma concentration after metabolite correction, to generate an arterial blood curve as input function for kinetic modeling.

The post-drug PET scan should be conducted with the identical acquisition parameters to those used for the baseline PET scan, with the only difference being test drug administration (***Fig. 2***).

#### PET image data processing

PET image data processing is the most skill-dependent task in PET clinical applications. Continuously acquired raw data need to be framed into multiple static images at various time intervals and transformed into sinograms. After reconstruction, these framed, sequential images reflect the spatial distribution of the tracer over time as a series of quantitative volumetric images. By defining certain regions of interest (ROI), radioactivity concentration in any given region can be measured on all image frames. By plotting the radioactivity within the ROI against time, the regional kinetics, or tissue pharmacokinetics, can be measured, generating what is called a time-activity curve.

### PET compartments

As mentioned above, the receptor occupancy [*O*(%)] calculation needs binding potential values of baseline PET and post-drug PET. Therefore, the majority of the effort for PET image analysis is spent obtaining binding potential values, either directly, or by binding potential = C_B_/C_P_.

**Fig. 5:**
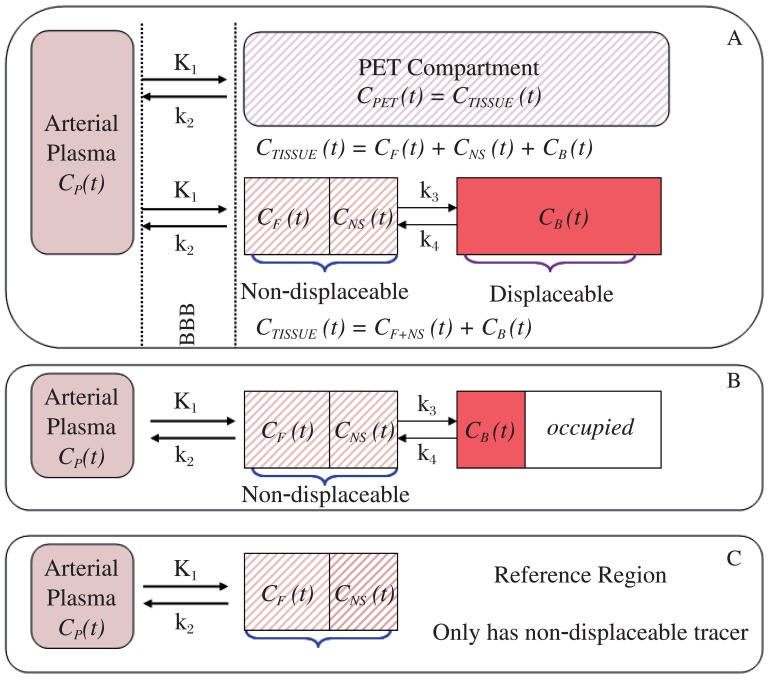
PET compartments in target (receptor-rich) region at baseline (A), post-drug with receptor occupancy (B), and in reference region void of target receptor (C).

To facilitate analysis, the tracer behavior *in vivo* is divided into virtual compartments. For PET brain imaging, only two sets of measures are available for extraction. One is the arterial plasma, obtained by measuring the tracer concentrations with the collected arterial blood samples as in conventional pharmacokinetics. The other is the “PET Brain Compartment” as shown in ***Fig. 5A***, which equals the total radioactivity concentration in brain tissue (C_T_) at time t expressed as C_PET_ (t) or C_TISSUE_ (t). C_TISSUE_ is the average concentration of a tracer that can be measured directly within a given ROI in the units of microcurie per cubic centimeter (µCi/cm^3^). C_TISSUE_ in an image acquired between the interval of t_1_ and t_2_ can also be mathematically expressed as: 
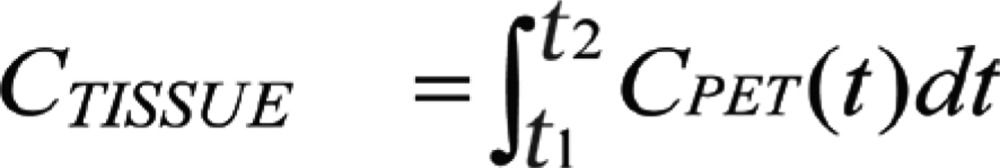


In any brain region, C_TISSUE_ is measured as the summed concentrations of free (C_F_), non-specific bound (C_NS_) and specific bound (C_B_), i.e.

*C_TISSUE_ (t)* = *C_F_ (t)* + *C_NS_* *(t)* + *C_B_* *(t)*

Because the free and non-specific bound compartments can be regarded as a whole when the two compartments can equilibrate rapidly[5], the above equation can be written as:

*C_TISSUE_ (t)* = *C*_*F*+*NS*_ *(t)* + *C_B_ (t) or*

*C_T_* = *C*_*F*+*NS*_ + *C_B_* and

*C_B_* = *C_T_* – *C*_*F*+*NS*_

In this situation, only the specific bound (C_B_) compartment is replaceable by a test drug, and the combined compartment C_F+NS_ is ubiquitous all over the brain, and cannot be reduced by a competitive drug, shown as non-replaceable in ***Fig. 5A***.

When the test drug is administered to compete with the receptor binding during the post-drug PET scan, only the specific bound fraction can be displaced (occupancy), and the sum of C_F_ and C_NS_ remain unchanged as the non-displaceable fraction (***Fig. 5B***). In some cases, there may be regions in the brain that are devoid of the target receptor (in which, C_B_ = 0). This area can be used as reference, and its concentration [C_R_] equals the non-replaceable fraction, i.e, C_R_ = C_F+NS_ (***Fig. 5C***).

### Binding potential calculation by modeling

From PET compartments shown in ***Fig. 5A***, a tracer in the arterial plasma penetrates blood brain barrier freely into PET compartment, within which some exist as free ligand, some bound specifically to its receptor, and some bound non-specifically. The tracer exchange is freely between these compartments until equilibrium is reached, and the movement and binding rates are linearly related to the difference in tracer concentrations of the two adjacent compartments as shown in the following differential equations[4]-[6]:

*dC*_*F*+*NS*_ *(t)/dt* = *K_1_C_P_(t)* – *(k_2_* + *k_3_)C*_*F*+*NS*_*(t)* + *k_4_C_B_(t)*

*dC_B_(t)/dt* = *k_3_C*_*F*+*NS*_*(t)* – *k_4_C_B_(t)*

Herein, K_1_ is the transportation rate, or the perfusion, as mL/(min·g); while k_2_, k_3_ and k_4_ are rate constants of the tracer as 1/min at time t (min).

At this stage, the readers can be easily misled into obtaining steady state C_B_ value by solving the above differential equations. In reality, the equilibrium is not easy to reach or identify. Therefore, instead of obtaining C_B_, the binding potential can be calculated directly as a combination of the kinetic parameters as “macro parameter” as:

Binding potential 
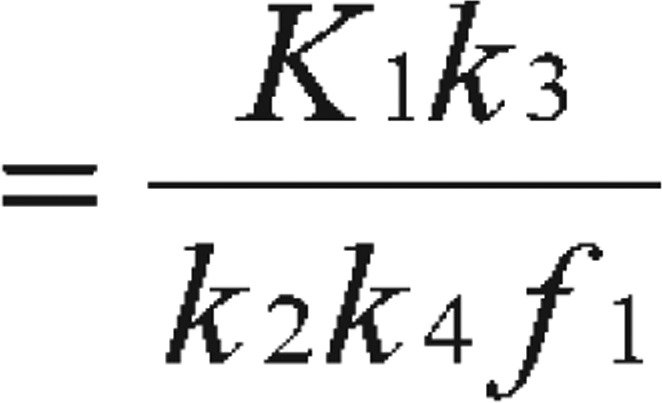
[5] or Binding potential 
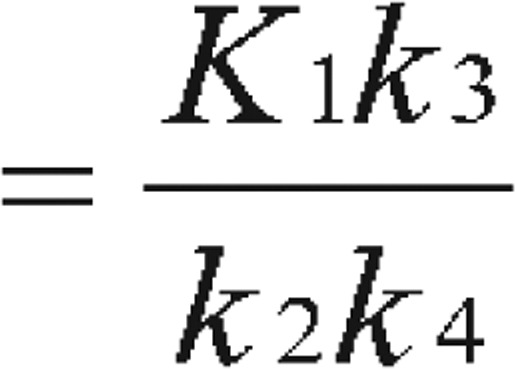
[6] or obtained as simply Binding potential 
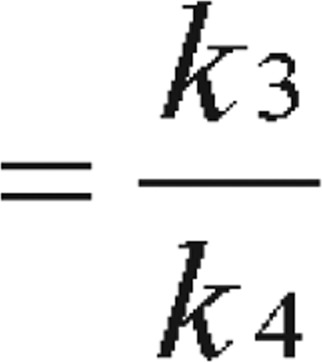
[2],[7],[8]

In this case, f1 is the free fraction of tracer in the plasma[7]. With the metabolite-corrected plasma time-activity curve as input function, the kinetic parameters (K_1_, k_2_, k_3_, and k_4_) of the receptor-rich areas can be derived by analyzing tissue time-activity curves with nonlinear regression using Levenberg-Marquart least-squares minimization procedure that can be implemented in MATLAB (The Math Works) or PMOD (PMOD Technologies) software packages[8],[9].

The compartmental modeling with arterial blood as input function is the most reliable and the classic method used in PET studies. It is especially useful in some cases, when there is no region devoid of the receptor available as reference region, either because the receptor has a disseminated brain distribution, or because the receptor is without well-characterization.

### Binding potential calculation by reference region and modified binding potential (BP′)

With reference region(s) available as in ***Fig. 5***, three additional methods are available for measuring binding potential. The distribution volume (DV) ratio method for the calculation of a modified binding potential (BP′):

BP′ = (DV_T_-DV_R_)/DV_R_ = DV_T_/DV_R_ –1[5].

DV is defined as the ratio of the tracer concentration in tissue to that in plasma at equilibrium, reflecting that the tracer is being concentrated in the tissue. DV_T_(= C_T_/C_P_) and DV_R_(= C_T_/C_P_) are values of distribution volume of the tracer in the target and in the reference region, respectively. By kinetic modeling, both DV_T_ and DV_R_ can be obtained without identifying equilibrium, by using graphical analysis of the ratios of tissue concentrations and plasma concentrations to extrapolate the observed data[10]. When an apparent steady state can be identified as “late-scan”, a simplified DV can be obtained by using the tissue tracer concentration obtained from a single static scan divided by the corresponding plasma parental tracer concentration[11].

A more attractive approach for obtaining binding potential is the simplified reference tissue model[12],[13]. Simplified reference tissue model uses time-activity curves of reference tissue to provide input function to the kinetic model. The advantage of the simplified reference tissue model is clear: no arterial cannulation and sampling are required, reducing the degree of invasiveness and the level of complexity of the PET study as a whole.

The most desirable method for calculating binding potential is using the tissue ratio method to calculate the PB as specific uptake ratio at pseudo-equilibrium period:

Specific uptake ratio = (C_T_ – C_R_)/C_R_[14] = C_T_/C_R_ – 1

The specific uptake ratio method can also be mathematically derived from the distribution volume ratio method[5]. With DV_T_ = C_T_/C_P_ and DV_R_ = C_R_/C_P_, the equation binding potential′ = (DV_T_ – DV_R_)/DV_R_ = DV_T_/DV_R_ –1 can be converted to modified binding potential (BP′)= [(C_T_/C_P_) – (C_R_/C_P_)]/(C_R_/C_P_) = C_T_/C_R_ – 1.

With modified binding potential (BP′) or specific uptake ratio, based on formula (1), a more practicable method is generated for calculation receptor occupancy [*O*(%)][1]: 
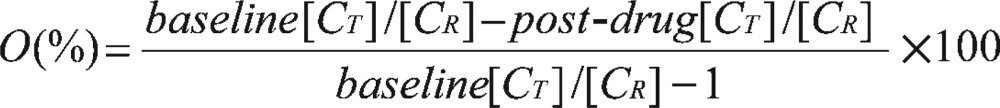
(2)

Evidently, the tissue ratio method is the simplest approach for obtaining binding potential because this method obviates the arterial blood sampling and avoids dynamic scan over a long acquisition time. Only a static image is required at a time frame where the specific uptake ratio, or the tissue ratio C_T_/C_R_ remains stable, i.e. taking the image during a pseudo-equilibrium interval[13]. The specific uptake ratio method is more often reported in studies with long half-life tracers, which can reach a stable and relatively long pseudo-equilibrium interval with single radioligand injection[13],[14]. For a new tracer, it will be wise to validate the specific uptake ratio against kinetic compartmental modeling in the first few subjects to identify the pseudo-equilibrium interval.

In general, the binding potential measurement in receptor occupancy studies is not as stringent as that in the standalone brain receptor imaging. The bias in the binding potential measurement can be cancelled off during receptor occupancy calculation provided the other parameters contributing to the binding potential (e.g. K_D_ or *f*_1_) do not change after the test drug administration[7].

### Receptor occupancy and drug dosing

The significance and value of receptor occupancy studies can be summarized into three tiers. Firstly, without wasting effort and time to label a drug candidate, we learn whether the test drug can penetrate the human blood-brain barrier. Secondly, superior to imaging the radiolabeled drug itself, receptor occupancy studies can tell whether the test drug has bound to its target-by-target engagement. Lastly, and most importantly, the receptor occupancy curve provides a direct quantitative measurement of the level of target engagement of the test drug at a given dose level. This will provide a great bridge for the parmacokinetics/pharmacodynamics relationship, connecting drug dose and drug efficacy. For example, when there is no efficacious benefit observed, the receptor occupancy curve can help determine whether the dose has not been pushed high enough to reach a certain occupancy level, or if receptor binding is simply not an effective target for the disease.

## CONCLUSION

Receptor occupancy with PET imaging is a powerful and robust tool for establishing the pivotal relationship between dose and pharmacological outcomes. By measuring the change in the binding potential reduced by a competitive test drug, receptor occupancy measurement should be an essential component of CNS drug development. However, these applications have been hindered by the confusion of nomenclature, the complexity of PB measurements, and receptor occupancy calculations[7]. We intend here to make it clear that obtaining the value of binding potential is key to receptor occupancy measurements. The binding potential can be measured by direct modeling using plasma tracer concentration as arterial input, or as an approximate value of binding potential′ or as specific uptake ratio, when a reference region devoid of target receptor is available and a pseudo-equilibrium interval can be identified. All of these calculations can be conducted using elementary algebra, and all equations written in calculus are simply descriptions of mathematical language.
